# HR-MS Based Untargeted Lipidomics Reveals Characteristic Lipid Signatures of Wilson’s Disease

**DOI:** 10.3389/fphar.2021.754185

**Published:** 2021-11-22

**Authors:** Yixiao Zhi, Yujiao Sun, Yonggeng Jiao, Chen Pan, Zeming Wu, Chang Liu, Jie Su, Jie Zhou, Dong Shang, Junqi Niu, Rui Hua, Peiyuan Yin

**Affiliations:** ^1^ Clinical Laboratory of Integrative Medicine, First Affiliated Hospital of Dalian Medical University, Dalian, China; ^2^ Department of Hepatology, The First Hospital of Jilin University, Changchun, China; ^3^ Institute of Integrative Medicine, Dalian Medical University, Dalian, China; ^4^ Department of Anesthesiology Jilin Province FAW General Hospital, Changchun, China; ^5^ Department of General Surgery, First Affiliated Hospital of Dalian Medical University, Dalian, China; ^6^ iPhenome biotechnology Inc. Dalian (Yun Pu Kang), Dalian, China

**Keywords:** Wilson disease, lipidomics, biomarkers, triglyceride, metabolomic

## Abstract

**Background and Aims:** The diagnosis of Wilson’s disease (WD) is challenging by clinical or genetic criteria. A typical early pathological change of WD is the increased liver lipid deposition and lowered serum triglyceride (TG). Therefore, the contents of serum lipids may provide evidence for screening of biomarkers for WD.

**Methods:** 34 WD patients, 31 WD relatives, and 65 normal controls were enrolled in this study. Serum lipidomics data was acquired by an ultra-high-performance liquid chromatography high-resolution mass spectrometry system, and the data were analyzed by multivariate statistical methods.

**Results:** Of all 510 identified lipids, there are 297 differential lipids between the WD and controls, 378 differential lipids between the relatives and controls, and 119 differential lipids between the patients and relatives. In WD, the abundances of most saturated TG were increased, whereas other unsaturated lipids decreased, including phosphatidylcholine (PC), sphingomyelin (SM), lysophosphatidylcholine (LPC), ceramide (Cer), and phosphatidylserine (PS). We also found many serum lipid species may be used as biomarkers for WD. The areas under the receiver operating characteristic curve (AUC) of PS (35:0), PS (38:5), and PS (34:0) were 0.919, 0.843, and 0.907. The AUCs of TG (38:0) and CerG1 (d42:2) were 0.948 and 0.915 and the AUCs of LPC (17:0) and LPC (15:0) were 0.980 and 0.960, respectively. The lipid biomarker panel exhibits good diagnostic performance for WD. The correlation networks were built among the different groups and the potential mechanisms of differential lipids were discussed. Interestingly, similar lipid profile of WD is also found in their relatives, which indicated the changes may also related to the mutation of the ATP7B gene.

**Conclusions:** Lipid deregulation is another important hallmark of WD besides the deposition of copper. Our lipidomic results provide new insights into the diagnostic and therapeutic targets of WD.

## Introduction

Wilson’s disease (WD) is an autosomal recessive genetic disease, which is due to a mutation of the ATP7B gene that leads to a copper metabolism disorder (Ala et al., 2007; Członkowska et al., 2018). WD presents most commonly between ages 5 and 35 (Xie and Wu, 2017). Copper ions are deposited in the liver, brain, cornea, kidneys and bone, which progressively aggravates organ damage (Cumings, 1948; Mounajjed et al., 2013; Bandmann et al., 2015). The most prominent clinical presentations of WD are liver disease and cirrhosis, neurologic symptoms and psychiatric features (Mulligan and Bronstein, 2020). Available treatments include zinc salts and chelators, which allow for sufficient control of symptoms. But these drugs do not cure the disease and develop severe side effects (Ranucci et al., 2017).

The diagnosis of WD is often delayed. There is no gold standard because of the nonspecific clinical features (Ryan et al., 2019). The diagnosis of the disease primarily depends on clinical manifestations, laboratory test results, decreases in ceruloplasmin content, increases in 24-h urine copper excretion, liver biopsy findings (increase in liver copper content) and ATP7B gene mutation (Steindl et al., 1997; Ferenci et al., 2019). When low serum ceruloplasmin content was used as a screening test for WD, its positive predictive value was only 6% (Ferenci, 2014). Twenty-four-hour urinary copper excretion may be lower or even normal in 16–23% of WD patients, especially in children and asymptomatic siblings (European Association for Study of Liver, 2012). Hepatic copper content >250 μg/g dry weight is an important indicator of WD, but liver biopsy is an invasive procedure (Ferenci et al., 2005). Comprehensive molecular genetic screening is expensive and difficult because of more than 700 possible mutations (Ferenci, 2005; Mak and Lam, 2008).

Defective ATP7B function results in pathological copper accumulation, which leads to hepatic steatosis and liver injury in WD (Stättermayer et al., 2015). Copper accumulation markedly alters lipid metabolism, and the lipid peroxidation system that produces free redicals, changes enzyme activity and inhibits mitochondrial functions. These changes have been reported in patients and animal models (Gerosa et al., 2019). Our clinical observations indicated that the serum TG levels in WD patients are generally lower than that in healthy controls. The mean TG level in WD patients was 0.85 mmol/L, whereas the level was 1.19 mmol/L in the control group (normal level: 0.28–1.8 mmol/L). Although the TG level is within the normal range for both groups, it remains significantly lower in WD patients. Performing lipidomics to analyze WD metabolic disturbance will contribute to the elucidation of WD diagnosis and understanding of the pathogenesis of WD.

Lipidomics emerged in 2003 as an approach to study the metabolism of the cellular lipidome and has greatly advanced in recent years (Blanksby and Mitchell, 2010). The development of mass spectrometry (MS) has accelerated this emerging discipline (Han et al., 2012). Clinical lipidomics provides a powerful tool to investigate the links between lipids and corresponding diseases, which will play a critical role in prevention, diagnosis, and potential therapies (Cortes et al., 2014; Zhang et al., 2018). Although the development of disease-specific biomarkers provides noninvasive diagnosis for various diseases, it remains a challenge to identify and develop lipid-based and disease-specific biomarkers (Loomba et al., 2015).

In this study, WD patients, their immediate family or relatives and healthy subjects were enrolled. An ultra-high-performance liquid chromatography high-resolution mass spectrometry (UHPLC-HRMS) based method was used to acquire the lipidomic data. We aimed to describe the metabolic deregulations of WD and identify the characteristics of lipids for the disease. These lipidomic findings may help to explore the mechanisms that change in lipid molecules, which may serve as potential diagnostic or therapeutic biomarkers for WD.

## Materials and Methods

### Participants

In this study, 34 WD patients (WD group) were recruited from the Department of Hepatology at the First Hospital of Jilin University from January 2012 to February 2019. The diagnosis of WD was made according to the 2012 European Society for Wilson’s Disease guidelines in this study (European Association for Study of Liver, 2012). Patients who had viral type B or C liver disease, drug-induced liver disease, alcoholic liver disease, autoimmune liver disease, overlap syndrome, hemochromatosis, original or secondary malignant tumor or were pregnant or lactating were excluded. Another group included 31 relatives, who were WD patients’ immediate family or relatives (WDIR group). The clinical and laboratory tests were normal in the WDIR. Sixty-five healthy controls (HC group) were matched for age and sex. This study was approved by the ethics committees of the First Hospital of Jilin University (No. 2019-346). After obtaining informed consent from patients or their legal guardians, clinical records and plasma were tested and analyzed.

### Lipid Extraction

Serum samples were collected from participants on the early morning after an overnight fast (12 h). Then the serum samples were immediately stored at −80°C. The serum samples were thawed at 4°C for 60 min and an aliquot of 50 μl sample was added into a 1.5 ml Eppendorf (EP) tube (Axygen, United States). Then, 250 μl methanol and 750 μl methyl tert-butyl ether (MTBE) were added to the samples, and the samples were vortexed for 5 min. Next, 250 μl of water was added to the mixture, and the samples were shaken using Rotational Incubator QB-128 (Kylin-Bell, China) at 60 rpm/min for 30 min at room temperature and then kept at 4°C for 30 min to promote separation. The samples were then centrifuged at 4°C and 13,000 ×g for 15 min. The upper (lipid extract) phase was quantitatively transferred to a 96-well plate, dried under reduced pressure (Labconco, United States), and stored at −20°C. Before analysis, the lipid extract was redissolved in 500 μl of acetonitrile/isopropanol solution and transferred to a tube for ultra-high-performance liquid chromatography-high-resolution mass spectrometry (UHPLC-HRMS) detection. A pooled quality control (QC) sample was prepared by mixing equal amounts of all the samples.

### Untargeted Lipidomics Analysis

Liquid chromatography-mass spectrometry (LC-MS) was performed on a UHPLC system coupled with a Q-Exactive mass spectrometer (Thermo Scientific, United States). Chromatographic separation was performed on an Accucore C30 column (Thermo Scientific Inc., United States, 2.6 μm, 100 mm), with a column temperature of 50°C, a mobile phase A (60% acetonitrile, 40% water, 10 mM ammonium formate and 0.1% formic acid) and mobile phase B (90% isopropanol +10% acetonitrile, 10 mM ammonium formate and 0.1% formic acid), a gradient elution (0–1 min 100% B, 1–6 min 100–50% B, 6–30 min 50–0% B, 30–38 min column washing and re-equilibration), a flow rate of 0.3 ml·min-1, and an injection volume of 5 μl.

Ionization conditions of MS were positive ion spray (ESI+) mode detection, spray voltage of 4000 V, sheath gas and auxiliary gas of 45 and 10 arb, heater temperature of 350°C, capillary temperature of 320°C, and S-Lens RF level of 50%. When the negative ion spray (ESI-) mode was detected, the spray voltage was adjusted to 3500 V, and the other parameters remained the same as those for ESI + mode.

The sample analysis was carried out in two steps. First, full-scan-data-dependent tandem MS (MS^2^) was performed on all QC samples, and the obtained primary and secondary MS data were used to identify lipid molecular structures. Then, all samples were tested by high-resolution first-order MS full-scan detection with positive and negative ionization switching, and the data obtained were used to determine the relative quantification of lipids.

MS parameters of the lipid molecular structure were full-scan data dependent MS^2^ data acquisition carried out in ESI+ and ESI- modes. The resolution ratio of the first-order MS was 70,000 full widths at half maximum (FWHM); the mass scanning range was 300–1,400 m/z; the automatic gain control threshold target was 1×10^5^; the maximum ion implantation time (IT) was 100 ms; the resolution ratio of secondary MS was 17,500 FWHM; the automatic gain control target was 10^5^; the maximum IT was 80 ms; the dynamic exclusion time was 5 s; the parent ion isolation window was 1.0 Da, and the loop count was 10. The MS fragmentation energies of the stepped normalized collision energy (NCE) of ESI+ and ESI- MS were 25% + 40 and 35%, respectively.

The parameters of full-scan analysis were as follows: ESI+ and ESI- ionization were switched in real time; the resolution ratio of the first-order MS was 70,000 FWHM; the mass scanning range was 300–1,400 m/z; and the automatic gain control target was 3×10^6^.

### Data Analysis

LipidSearch software (Thermo Scientific, United States) was used to process the ddMS^2^ lipidomics data collected by UPLC-HRMS, including peak detection and lipid structure identification. The main setting parameters were as follows: The retrieval accuracy of primary class parent ion MS was 5 ppm, and the secondary fragment mass spectrum was 5 mDa.

For peak integration and relative quantification of lipid molecules, the qualitative list of lipids produced by LipidSearch software was imported into TraceFinder software (Thermo Scientific, United States) for peak integration. The obtained peak area was used for analyzing lipid relative quantification, and finally, a data matrix containing fragment ion information was output. The missing values in the data matrix were processed using the 80% devaluation principle. Then, the data were imported into Metaboanalyst 4.0 online software (https://www.metaboanalyst.ca/) and SIMCA-P 14.1 (Umetrics, Sweden) for mode discrimination analysis. Principal component analysis (PCA), partial least squares-discriminant analysis (PLS-DA), and orthogonal PLS-DA (OPLS-DA) were used to construct a group-based model and discover differentially abundant metabolites between groups. The false discovery rate (FDR; adjusted *p* < 0.05) and fold change (FC; adjusted *p* < 0.05) were obtained by univariate T-test and ANOVA, and a volcano map was drawn to find differentially abundant metabolites. The obtained differentially abundant metabolites were uploaded to Metaboanalyst.

Normal distribution of the quantitative data was confirmed by independent sample t-tests and was expressed as the mean ± SD. For each independent metabolite, the receiver operating characteristic (ROC) curve was used to calculate the area under the curve (AUC), 95% confidence interval (95% CI), cutoff value, sensitivity and specificity to evaluate the predictive value of each metabolite. For the combined indicators, logistic regression analysis and ROC curve analysis were used to calculate the AUC and the 95% CI. In this study, a two-sided test was used, and differences with *p* < 0.05 were considered statistically significant.

## Results

### Study Design and Data Acquisition

**FIGURE 1:**
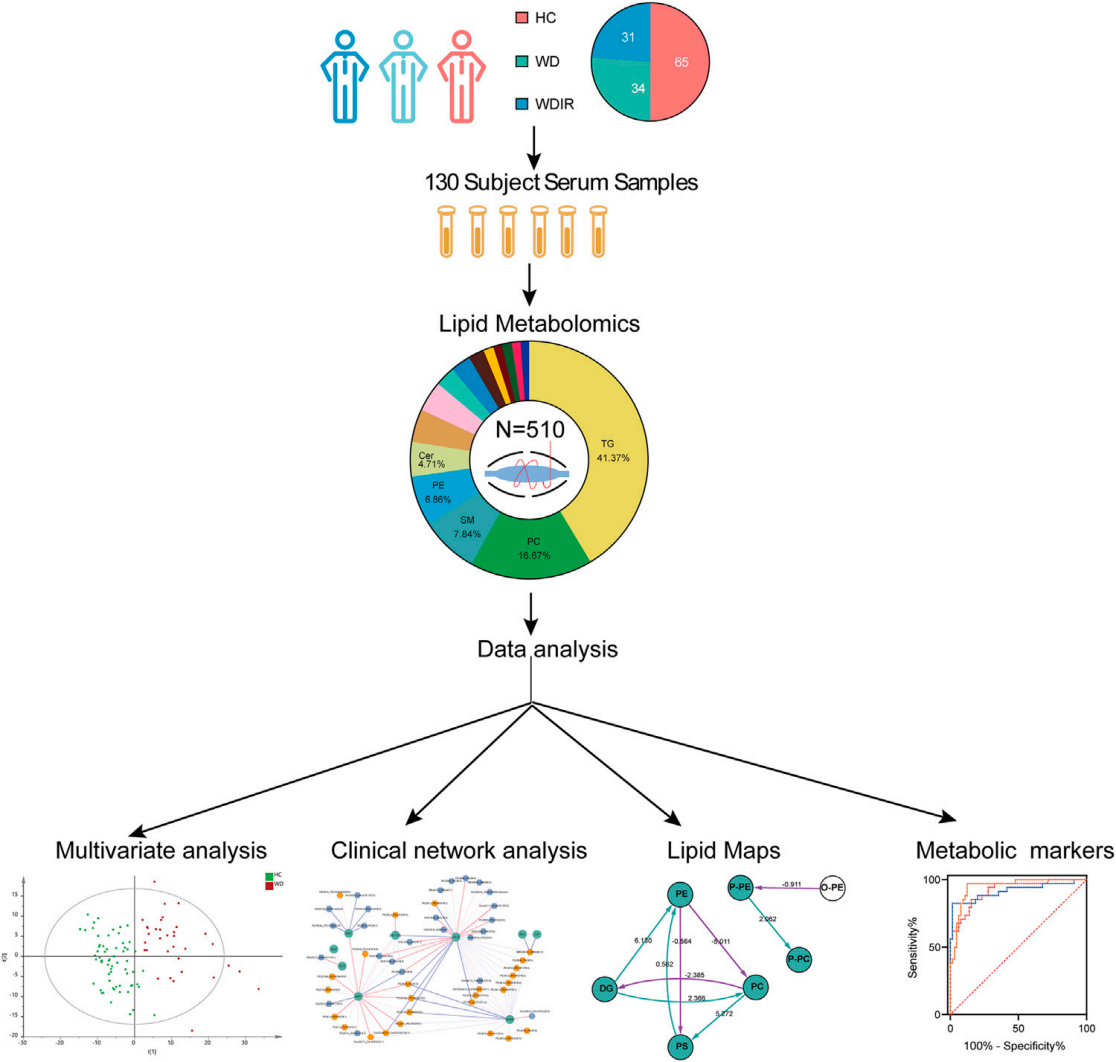
Workflow of WD serum analysis. This figure depicts the design and steps of this study, beginning with sample collection for the three cohorts, followed by untargeted lipidomic analysis, and then the various data analyses employed, including PCA, OPLS-DA, univariate T-test and ANOVA.

The flow diagram of this research design is illustrated in Figure 1. A total of 130 serum samples (from 65 healthy controls, 34 WD patients, and 31 WD patients’ immediate family or relatives) was analyzed by lipidomics to reveal lipid profiles in WD, followed by preprocessing of metabolomics data, including peak detection, alignment, filtering and normalization and then various statistical analyses were performed, including PCA, OPLS-DA, univariate T-test and ANOVA. Our lipidomics profiling identified 512 lipids, of which 510 passed QC procedures and were eligible for analysis (Table 1). 89.6% peaks had coefficients of variation (CV) below 10% and the CV value of 98.8% of the lipids was less than 30%, indicating that this experiment had good quantitative accuracy. (Supplementary Figure S1).

**TABLE 1:** Baseline characteristics of subjects.

Characteristics	Patient (n = 34)	Control (n = 65)
Age (years)	27.5 ± 10.9	29.3 ± 11.1
Sex (male/female)	16/18	36/29
Manifestation (cerebral/hepatic/mixed)	6/16/12	—
Ceruloplasmin (g/L)	0.1 ± 0.04	—
Serum copper level (μmol/L)	5.8 ± 3.8	—
Urinary copper level (μmol/24 h)	10.1 ± 26.8	—
Acetylcholine esterase (U/L)	5048.2 ± 2360.1	—
Alanine aminotransferase (U/L)	55.8 ± 96.1	18.5 ± 11.0
Aspartate aminotransferase (U/L)	45.5 ± 47.2	21.4 ± 5.5
Gamma-glutamyl aminotransferase (U/L)	54.2 ± 43.8	20.0 ± 11.2
Alkaline phosphatase (U/L)	99.6 ± 63.7	79.3 ± 51.7
Albumin (g/L)	36.9 ± 8.1	46.6 ± 2.7
Triglyceride (mmol/L)	0.8 ± 0.4	1.2 ± 0.4

Data are presented as mean as the mean ± SD.

### Clinical Profiles

WD patients were at the age of 8–50, with a large age span. The sex ratio of 1:1 conformed to the patterns of autosomal inheritance and the epidemiological characteristics of the disease. The most common clinical manifestations were single-system involvement; 52% of adolescents had hepatic involvement, whereas 28% had multisystem involvement, and middle-aged patients had mostly multisystem involvement. The concentration of serum ceruloplasmin (normal value: 0.2–0.5 g/L) less than 0.1 g/L is considered as strong evidence for diagnosis of WD (Bandmann et al., 2015). Ceruloplasmin content was significantly reduced (less than 0.1 g/L) in most patients, but 21% of patients still had a mildly reduced level of ceruloplasmin between 0.1 and 0.2 g/L. These patients were distributed in the adolescent stage, and 2/3 of them had liver-type symptoms. Serum TG levels were tested in patients, with an average value of 0.85 mmol/L (normal value: 0.28–1.8 mmol/L). Serum TG levels in controls were tested, with an average value of 1.19 mmol/L and generally higher than those of the patients. A T-test was performed for the differences between the two groups, with *p* < 0.05. The clinical details of the WD patients are shown in Table 1.

### Differential Lipids Analysis

**FIGURE 2:**
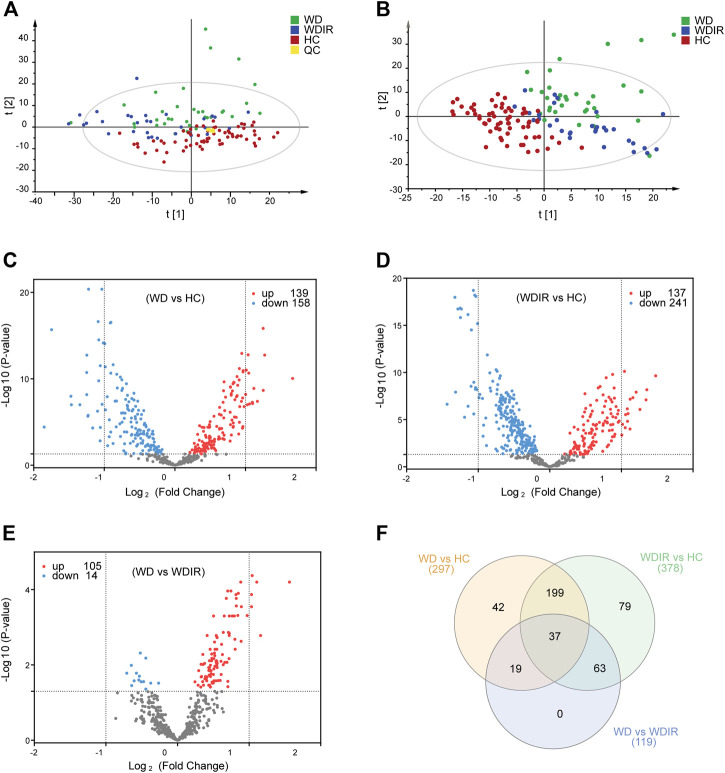
Overview of differential lipid profiles in three groups. **(A)** Principal component analysis (PCA) was used to test the samples of the WD, WDIR and HC (R2X = 0.893, Q2 = 0.772). **(B)** Partial least squares discriminant analysis (PLS-DA) was used to cluster the samples of the three groups (R2X = 0.524, R2X = 0.676, Q2 = 0.506). **(C)** A volcano plot showing the dysregulated features between WD and HC (Student’s t-test, FDR<0.05). **(D)** A volcano plot for the different lipids of WDIR and HC. **(E)** A volcano plot for the WD and WDIR. **(F)** Venn diagram displaying the number of differentially abundant metabolites that overlapped in the WD versus HC comparison (WD vs HC), WD versus WDIR comparison (WD vs WDIR), and WDIR versus HC comparison (WDIR vs HC).

SIMCA-P 14.1 software was used to model the lipidomic data of 130 serum samples. A PCA model was established for all participants **(** Figure 2A). The QC samples were clustered tightly together, and HC, WDIR, and WD could be clearly distinguished, which showed a trend of intergroup separation on the score plots. Given that WD is a genetic disease, the intersection between WD and WDIR conforms to genetic regulations. The results of the PLS-DA method showed better separation into separate clusters (Figure 2B). Furthermore, PLS-DA model validation with permutation tests (999 times) reflected that the metabolic alteration in each score plot was reliable and had clinical prediction significance (Supplementary Figure S2). A Venn diagram displayed the overlap of the differential metabolites in WD vs HC and WDIR vs HC (Figure 2F).

### Differential Lipids Between Wilson’s Disease and Healthy Controls

**FIGURE 3:**
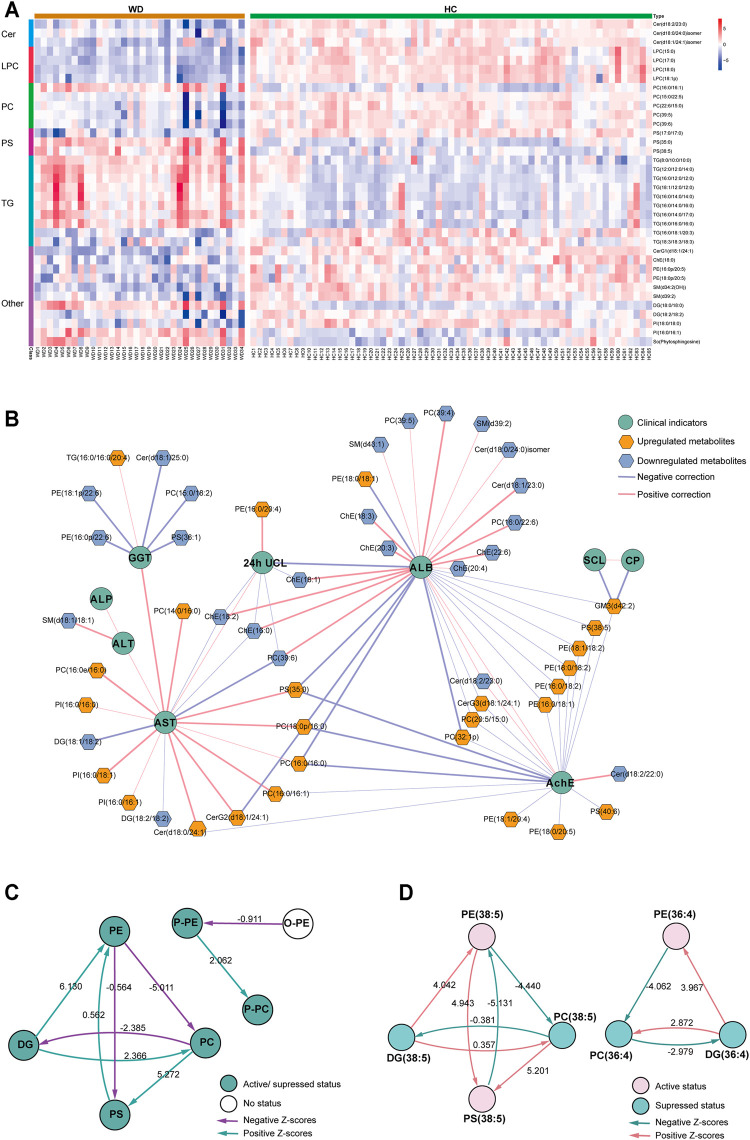
Metabolomic profiles differ between WD and HC. **(A)** Heat map of the 36 differential lipids between WD and HC. **(B)** Correlation network of differential metabolites and clinical indicators in WD and HC. The connections between two nodes were established by Pearson correlation (Student’s t-test, FDR <0.05). CP = ceruloplasmin; SCL = serum copper level; 24-h UCL = 24-h urinal copper level; ALT = alanine transaminase; AST = aspartate transaminase; GGT = γ-glutamyltransferase; ALP = alkaline phosphatase; AchE = acetylcholine esterase; ALB = albumin. **(C)** Lipid subclass correlation network of differentially abundant metabolites in WD and HC. **(D)** Lipid molecular species correlation network of differentially abundant metabolites in WD and HC.

We further analyzed the relationship between WD and HC. PLS-DA was used to classify the two groups, and clear separation between the two groups (Supplementary Figure S2). 297 differential lipids were identified (Figure 2C) which mainly included TG, phosphatidylcholine (PC), sphingomyelin (SM), lysophosphatidylcholine (LPC), ceramide (Cer), and phosphatidylserine (PS). Differences in metabolites were observed between WD and HC: 14 metabolites (Figure 3A) were significantly increased with an absolute log2 FC ≥ 1 and FDR<0.05 and 22 significantly downregulated (Figure 3A) with absolute log2 FC ≤ 1 and FDR<0.05. A heat map shows the 36 significantly differentially abundant metabolites (Figure 3A). The ultra-long-chain ceramides, phosphatidylethanolamine (PE) and LPC were all downregulated compared with HC. PC was mainly decreased, while TG was mainly increased. Eight TGs with saturated fatty acids (TG(8:0/10:0/10:0), TG(12:0/12:0/14:0), TG(16:0/12:0/12:0), TG(18:1/12:0/12:0), TG(16:0/14:0/14:0), TG(16:0/14:0/16:0), TG(16:0/14:0/17:0), TG(16:0/16:0/16:0)) were significantly increased (Figure 3A), and two TGs with unsaturated fatty acids [TG(16:0/18:1/20:3), TG(18:3/18:3/18:3)] were significantly decreased (Figures 3A, Figure 5H,I) in WD patients compared with healthy controls. PC is mainly distributed on the outer side of the cell membrane. In our results, the unsaturated PC (Figure 3A) showed a downward trend. PE is mainly distributed in the inner membrane of the cell membrane. In our results, the acyl structure of PE (PE(16:0/18:1), PE(16:0/18:2), PE(18:0/18:1), PE(18:0/18:2), PE(18:1/18:2), PE(18:1/20:4), PE(18:0/20:5)) was increased, while the acetal structure of PE [PE(16:0p/20:5), PE(16:0p/22:6), PE(18:1p/22:6), PE(18:0p/20:5)] was decreased (Figures 3A,B). Reduction in acetal PE content causes the destruction of the mitochondrial membrane (Maeba and Ueta, 2003), increases the generation of oxygen free radicals, causes mitochondrial dysfunction and energy metabolism failure, and causes cell apoptosis and even focal necrosis (Zoeller et al., 2002).

A correlation network based on the data of significantly differential metabolites and clinical factors in WD and HC revealed a change in the metabolic profile in WD patients (Figure 3B). Metabolites associated with γ-glutamyltransferase (GGT) or albumin (ALB), such as Cholesterol ester (ChE) and Cer, were mainly decreased, and the metabolites associated with aspartate transaminase (AST) and acetylcholine esterase (AchE), such as PC and PE, were mainly increased. Alkaline phosphatase (ALP), alanine transaminase (ALT), serum copper level (SCL) and ceruloplasmin had low correlations with differentially abundant metabolites in WD vs HC. Metabolic pathway analysis was conducted with BioPAN (Gaud et al., 2021) to further explore the metabolite–metabolite correlation between WD and HC (Figures 3C,D). The lipid-class active reactions were PC→PS→PE, DG→PE→PS, DG→PC and P-PE→P-PC. The lipid-class suppressed reaction chains were PE→PC→DG and PC→DG (Figure 3C). The synthesis of PS (38:5) was active, whereas decomposition was suppressed. The synthesis of DG (36:4) was suppressed, whereas decomposition was active (Figure 3D). Figure 3D indicates why PS (38:5) was a significantly increased metabolite and why DG (36:4) was a significantly decreased metabolite, as shown in Figure 3A.

### Differential Lipids Between WDIR and Healthy Controls

**FIGURE 4:**
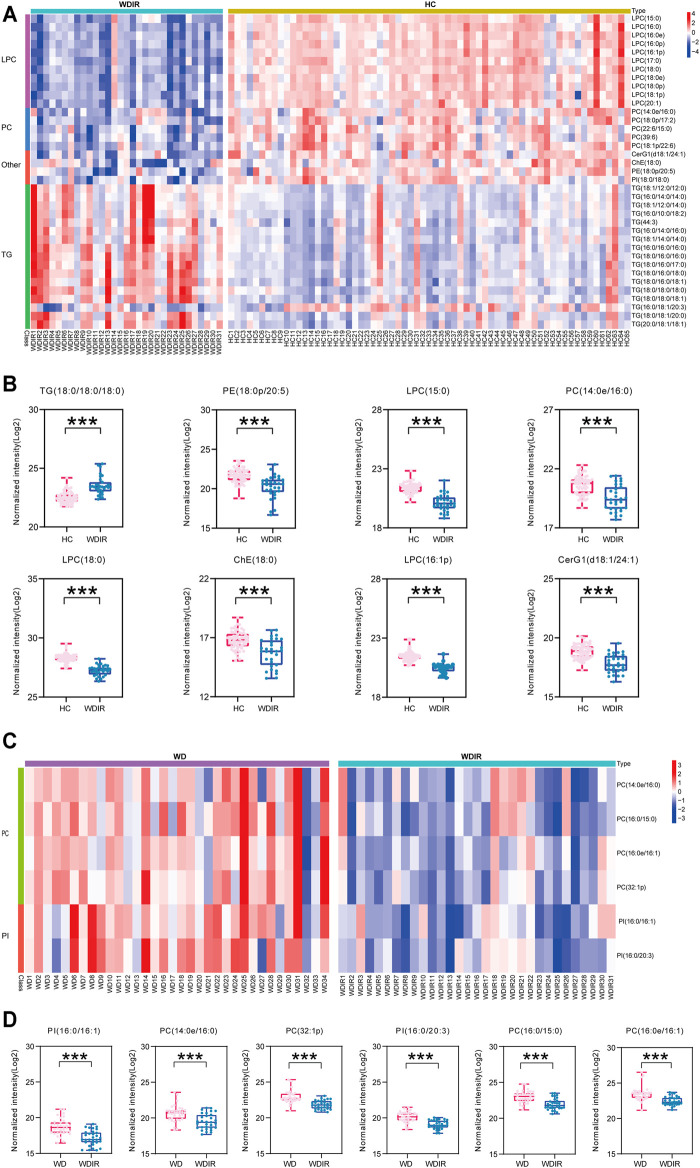
Metabolomic profiles differ between WDIR vs HC and WD vs WDIR. **(A)** Heat map of the 37 differential metabolites between WDIR and HC. **(B)** Relative concentration of differential metabolites screened by LASSO in WDIR and HC. **(C)** Heat map of the 6 differential metabolites between WD and WDIR. **(D)** Relative concentration of differential metabolites in WD and WDIR.

To explore the metabolic differences between WDIR and the HC, a PLS-DA model was established. As is shown in Supplementary Figure. S2C, a clear separation was observed between WDIR and HC. A total of 378 dysregulated metabolic features were discovered between the two groups, including 16 significantly up-regulated lipids and 21 significantly decreased lipids (Figure 2D). Figure 4A shows the 37 significantly differential lipids between WDIR and HC. Among the top 37 lipids, those decreased included PC and LPC. In contrast, the top metabolites that increased were mostly TGs. According to LASSO regression selection (Supplementary Figure S3), there were 7 metabolites for which the levels were significantly changed in WDIR compared to those in the controls, including PE (18:0p/20:5), PC (14:0e/16:0), ChE (18:0), LPC (15:0), LPC (18:0), LPC (16:1p), and CerG1(d18:1/24:1) (Figure 4B). Moreover, the level of TG (18:0/18:0/18:0) was found to be significantly elevated in WD patients’ immediate family or relatives. Interestingly, LPC (15:0) and CerG1(d18:1/24:1) were also decreased in WD patients compared with controls.

### Differential Lipids Between Wilson’s Disease and WDIR

Then, the differences between WD and WDIR were analyzed. A PLS-DA model was set up to show the overall metabolic differences between the two groups. The model demonstrated remarkable separation between WD patients and their relatives (Supplementary Figure S2). A total of 119 dysregulated metabolic features were identified between the two groups, including PC, SM, PI and TG (Figure 2E). The heat map showed that PC and PI were significantly upregulated in WD patients (Figure 4C). The levels of 6 significantly increased metabolites were higher in WD than in HC (Figure 4D).

### Potential Lipid Biomarkers for Wilson’s Disease

**FIGURE 5:**
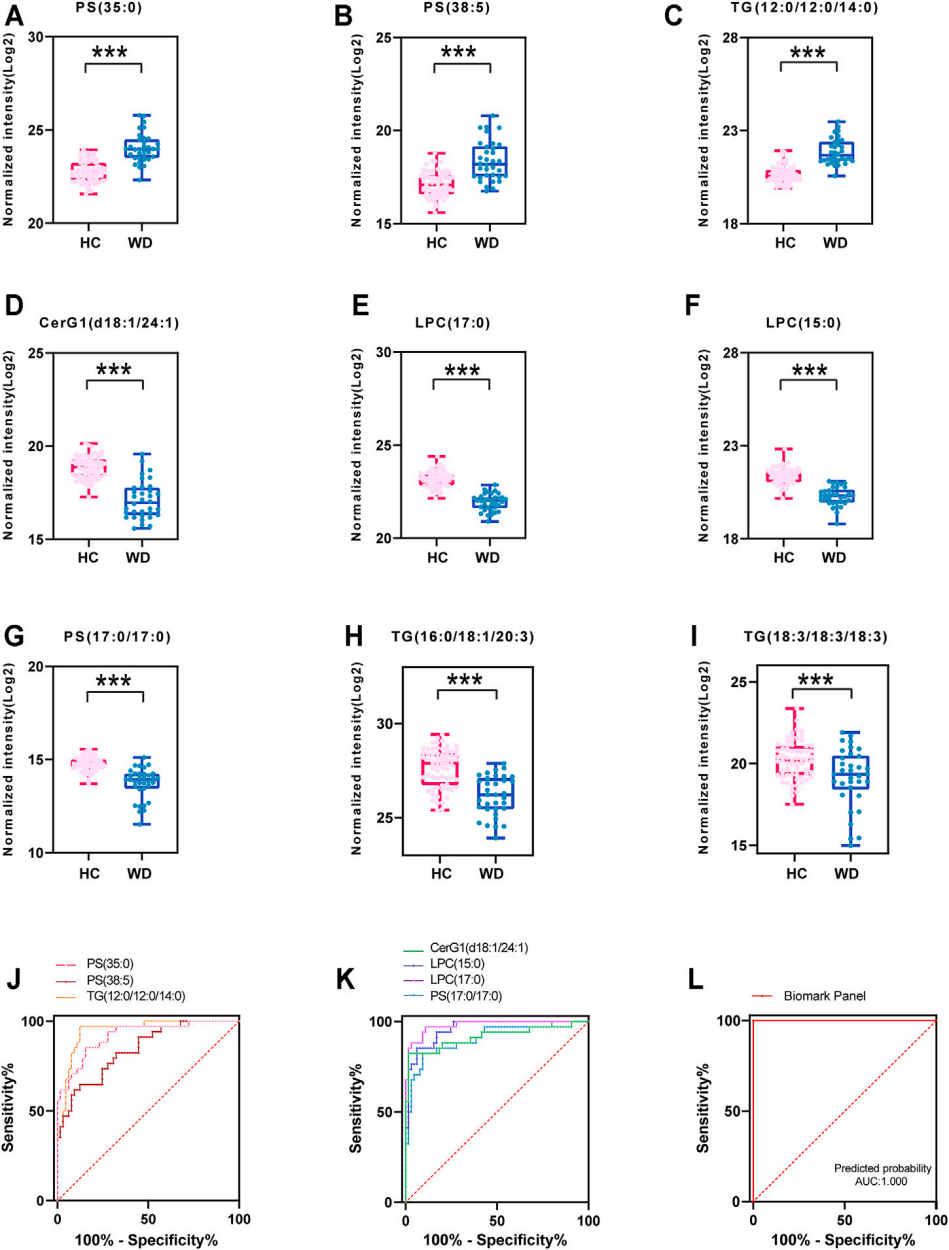
Fitting prediction of WD biomarkers. **(A–G)** Relative concentration of significantly differentially abundant metabolites screened by LASSO in WD and HC. **(H–I)** Relative unsaturated TG concentration of significantly differentially abundant metabolites in WD and HC. **(J–K)** ROC curve analysis of increased metabolites in WD and HC. **(L)** ROC curve analysis of the biomarker panel in WD and HC.

Seven important lipids were selected via least absolute shrinkage and selection operator (LASSO) regression significantly contributed to the diagnostic value for WD (Figures 5A–G, Supplementary Figure S3). The ROC curve was plotted for the 7 lipids to discriminate the WD patients and healthy controls. Among them, the AUC of the 7 lipids was greater than 0.8, with high sensitivity and specificity. PS (35:0) (95% CI: 0.862-0.976), PS (38:5) (95% CI: 0.763-0.922) and TG (38:0) (95% CI: 0.905-0.990) were increased differentially abundant metabolites (Figure 5J). CerG1(d42:2) (95% CI: 0.844-0.987), LPC(15:0) (95% CI: 0.926-0.993), LPC (17:0) (95% CI: 0.958-1.000) and PS (34:0) (95% CI: 0.842-0.972) were decreased (Figure 5K). LPC (17:0) and LPC (15:0) were shown to have the greatest chance of appearing in the biomarker panel. Binary Logistic regression analysis was performed on the biomarker panel and the AUC is 1.000 (Figure 5L).

## Discussion

WD occurs in siblings (25%) but rarely in the previous generation (0.5%) or offspring (0.5%) because of the autosomal recessive mode of transmission (Brunet et al., 2012). The patients’ family members may have late onset and be asymptomatic and may have different phenotypes even with the same genotype. Important diagnostic indicators for WD, including ceruloplasmin, 24 h urine copper excretion, Kayser Fleischer (KF) ring, and hepatic copper concentration, are all limited and not appropriate for screening in populations or newborns (Członkowska et al., 2018). Despite the potential devastating course of WD, the time of onset is not predictable. Thus, we aimed to identify potential lipid biomarkers that can provide an early warning for WD onset. Early diagnosis of WD is crucial for effective treatments that can prevent many manifestations of WD.

At the early stages of the disease, copper accumulation in the liver has a major effect on the dysregulation of lipid metabolism (Huster et al., 2007). In our study, there were common changes in TG molecules in WD patients and their relatives compared to those in controls. There were 297 differential lipids between WD and HC, of which TG, PC, SM, PE and LPC accounted for the majority. TG molecules except TG (16:0/18:1/20:3) were significantly upregulated in WDIR compared with HC. The detected TG molecules among the differential metabolites between WD and HC mostly had saturated fatty acid chains. However, only a few TGs containing unsaturated fatty acid chains were decreased in WD.

In the human body, most of the total TG has unsaturated fatty acid chains (Kawano and Cohen, 2013). Therefore, when testing the total TG content in serum, the change would be consistent with most TGs containing unsaturated fatty acid chains showing a downward trend. In this study, although the serum TG contents of WD (0.85 mmol/L) were at lower range of normal, there is still a significant decrease compared with the controls (1.19 mmol/L), clearly indicative of lipid metabolism disorder with pathology in the liver in which fat droplets are deposited (Lutsenko, 2014). The extent of the accumulation of these saturated fatty acids in the steatotic liver parallels the liver disease severity in nonalcoholic steatohepatitis (Chiappini et al., 2016; Zhou et al., 2016; Chiappini et al., 2017). We speculated that hepatic steatosis was related not only to the amount of lipids but also to their specific composition and proportion. The accumulated copper in WD is assumed to lead to the development of chronic hepatitis by stimulating the production of reactive oxygen species and accelerating the formation of harmful hydroxyl radicals (Du et al., 2004). Unsaturated fatty acids containing multiple double bonds play a protective role in liver by redox reaction and reaction with free radicals (DeLany et al., 2000; Barber et al., 2021). Animal studies suggest PUFAs could reduce hepatic TG deposition (Sekiya et al., 2003). In our study, saturated fatty acids were upregulated and unsaturated fatty acids were downregulated in WD patients, resulting in a reduction of hepatoprotective effect and WD exacerbations. Unsaturated fatty acids were upregulated in their asymptomatic relatives. We speculated that unsaturated fatty acids could protect the liver against lipid accumulation.

It is well-known that dietary fat, along with adipose tissue lipolysis and hepatic *de novo* lipogenesis, affects hepatic lipogenesis (Luukkonen et al., 2018). There is evidence that saturated fatty acids derived from the diets may influence liver fat content (Allard et al., 2008; Petersson et al., 2010). Liver TGs may be derived from the plasma or be newly synthesized from glucose (Kawano and Cohen, 2013). Consumption of low glycemic index diets could improve liver lipid metabolism and disease prognosis, since glucose promotes lipogenesis by activation of carbohydrate response element binding protein (ChREBP) (Kawano and Cohen, 2013). Additionally, dietary (n-6) and (n-3) PUFAs are potent inhibitors of hepatic lipogenesis (Jump, 2011). But n-3 PUFAs cannot be synthesized by the human body and must be extracted from exogenous food (fish oil, flax seeds, etc.). Our results implied that the imbalance of intrahetapic lipid in WD patients could be corrected by supplementation with unsaturated fatty acids, since dietary PUFAs are able to regulate hepatic glycolysis and *de novo* lipogenesis and to limit TG deposition in the liver (Jump, 2008; Di Minno et al., 2012). Dietary supplementation with unsaturated fatty acids, especially PUFAs, and consumption of low glycemic index diets before the onset of WD may be of for protecting liver and delay the progression of cirrhosis. Further work is warranted to understand the role of unsaturated fatty acids in WD pathogenesis and therapeutics.

The result that LPCs were downregulated in both WD patients and WDIR compared with HC is intriguing. To date, it has been found that LPC is decreased in drug-induced liver injury, viral hepatitis, alcoholic hepatitis, nonalcoholic fatty liver, liver failure and liver cirrhosis (Huang et al., 2013; Saito et al., 2014; Sherriff et al., 2016). However, there is no report on the pathogenic mechanisms of LPCs in WD. The mechanisms underlying LPC lipotoxicity include lipoapoptosis triggered by c-Jun NH2-terminal kinase (JNK) and endoplasmic reticulum (ER) stress activation, causing mitochondrial dysfunction, and LPC impairs hepatic mitochondrial oxidative phosphorylation, inducing hepatocyte lipoapoptosis (Kakisaka et al., 2012; Hollie et al., 2014). Most of these lipotoxic mechanisms overlap those of saturated fatty acids, suggesting that LPC depletion could be a major downstream effector of saturated fatty acid toxicity.

Our study showed that the levels of PS (35:0) and PS (38:5) increased, whereas the levels of PS (34:0) decreased in WD patients. PS, accounting for 13–15% of the phospholipids in the human cerebral cortex, is an important precursor for the two major phospholipids PE and PC (Kim et al., 2014). It has also been found that PS can reduce oxidative stress in the brain and stimulate neurotransmitter release (Suzuki et al., 2001; Chaung et al., 2013). The significantly differential abundance of PS may be associated with the neurological presentation in WD. The lack of ATP7B-mediated hepatic efflux of copper contributes to the failure of mitochondria to handle massive copper accumulation (Zischka and Lichtmannegger, 2014; Polishchuk et al., 2019). Disruption of ER-mitochondrial PS transfer is a newly reported new mechanism involved in the development of liver disease (Hernández-Alvarez et al., 2019). It has also been found that PS on blood cells and endothelial cells plays an important role in the hypercoagulable state in cirrhotic patients (Wu et al., 2016).

We found a significant decrease of ultra-long-chain ceramides and glycosphingolipids. Ceramides containing long side chains such as palmitic (CER 16:0) and stearic (CER 18:0) are suspected to be linked to hepatic steatosis (Wasilewska et al., 2018). Cer-derived C22:0-24:0 ceramides are crucial for regulating hepatic function. C16:0 ceramides is suspected to correlate with hepatosteatosis (Turpin et al., 2014). Meanwhile, ceramide can induce hepatocyte apoptosis in WD patients, which is an important cause of hepatocyte loss (Lang et al., 2007; Engin, 2017). It is well known that increased and uncontrolled death of hepatocytes results in hepatic steatosis and cirrhosis, which are the most commonly described dysfunctions in WD (Wooton-Kee et al., 2020). Previous studies have shown that ceramide content decreased in the early stages of the disease and increased when liver cell apoptosis begins to exhibit pathological changes, such as liver cirrhosis. The reduction in Cer-mediated mitochondrial division may product the mitochondrial fatty acid oxidation capacity, which is almost certainly key to reducing hepatic steatosis (Samuel and Shulman, 2019). For a potential application of WD diagnosis, we established a biomarker panel that comprised 7 lipids (PS(35:0), PS (38:5), TG (38:0), CerG1(d42:2), LPC (17:0), LPC (15:0), and PS (34:0)). However, there are still some limitations in our study. Since WD is a rare disease, only 34 patients from one medical center were enrolled in our study during 7 years of sample collection. The diagnostic performance of the lipid biomarkers requires further validations in a larger cohort and a multiple-center study.

## Conclusion

Based on our lipidomics data, we found an interesting metabolic profile of WD patients. Our results highlight lipids deregulations with the deposition of copper. The abnormal lipid metabolism provides possible biomarkers for the diagnosis of WD, as a complementary of ceruloplasmin. More importantly, lipid metabolism may also be an effective therapeutic target for WD, which may alleviate the hepatic steatosis and liver injury among the patients.

## Data Availability

The original contributions presented in the study are included in the article/Supplementary Material, further inquiries can be directed to the corresponding authors.
