# Supplementary figures and images for: HR-MS Based Untargeted Lipidomics Reveals Characteristic Lipid Signatures of Wilson’s Disease
Source: Front Pharmacol. 2021 Nov 22;12:754185. doi: 10.3389/fphar.2021.754185 (PMC8645799; doi:10.3389/fphar.2021.754185)

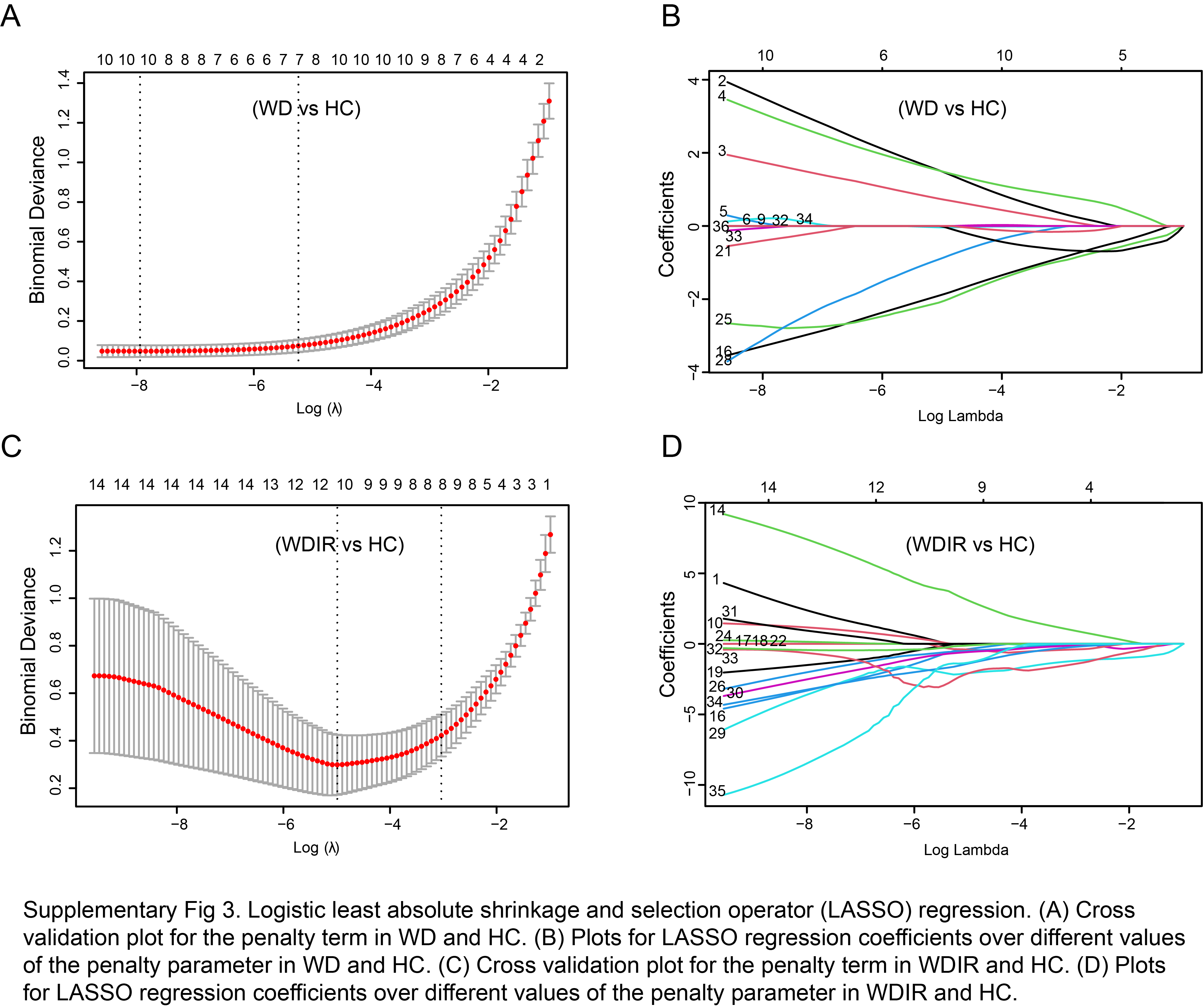

Supplement: Supplementary file 1 [file Image3.TIF]

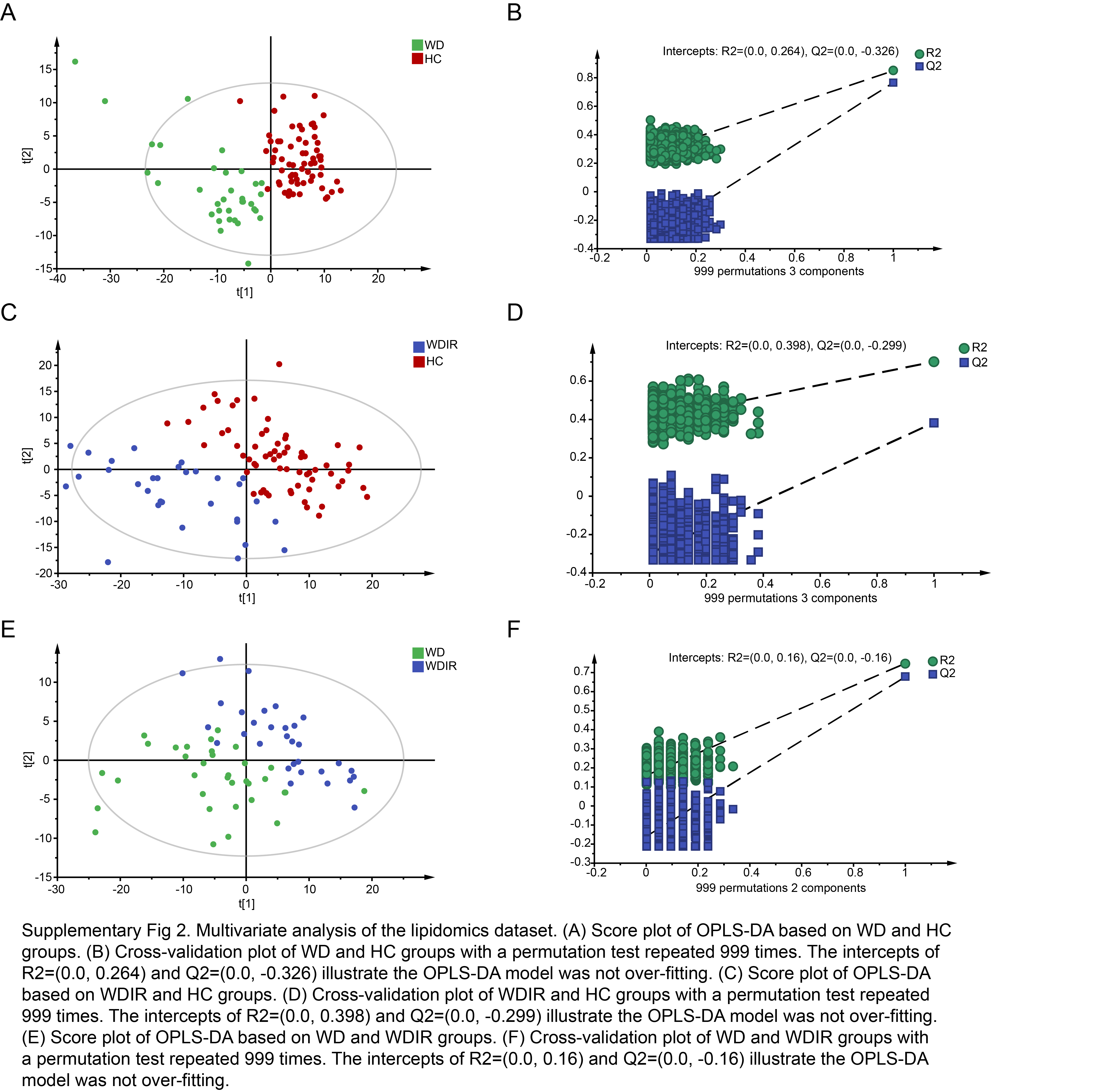

Supplement: Supplementary file 2 [file Image2.TIF]

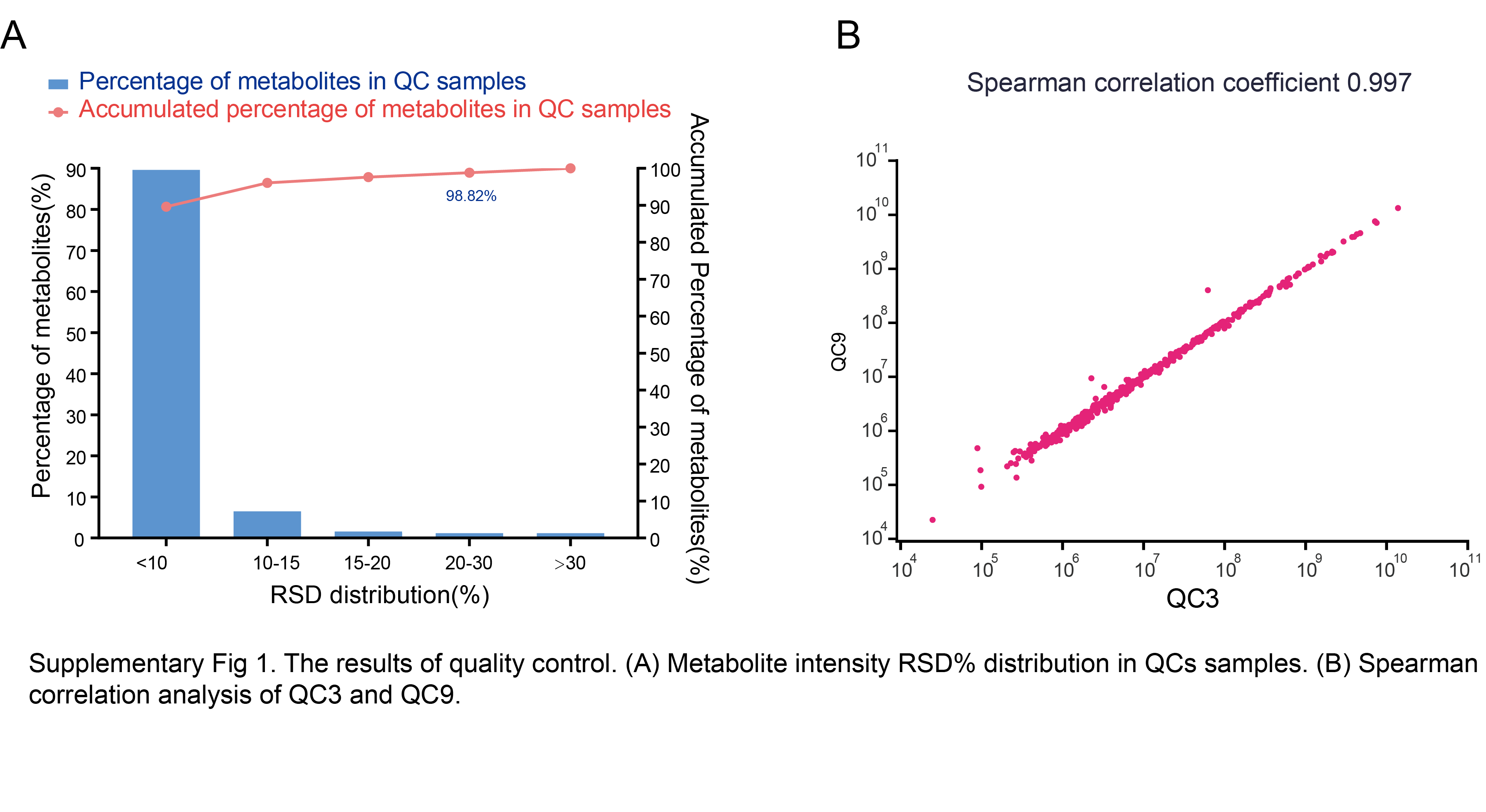

Supplement: Supplementary file 3 [file Image1.TIF]
